# The FORCIS database: A global census of planktonic Foraminifera from ocean waters

**DOI:** 10.1038/s41597-023-02264-2

**Published:** 2023-06-03

**Authors:** Sonia Chaabane, Thibault de Garidel-Thoron, Xavier Giraud, Ralf Schiebel, Gregory Beaugrand, Geert-Jan Brummer, Nicolas Casajus, Mattia Greco, Maria Grigoratou, Hélène Howa, Lukas Jonkers, Michal Kucera, Azumi Kuroyanagi, Julie Meilland, Fanny Monteiro, Graham Mortyn, Ahuva Almogi-Labin, Hirofumi Asahi, Simona Avnaim-Katav, Franck Bassinot, Catherine V. Davis, David B. Field, Iván Hernández-Almeida, Barak Herut, Graham Hosie, Will Howard, Anna Jentzen, David G. Johns, Lloyd Keigwin, John Kitchener, Karen E. Kohfeld, Douglas V. O. Lessa, Clara Manno, Margarita Marchant, Siri Ofstad, Joseph D. Ortiz, Alexandra Post, Andres Rigual-Hernandez, Marina C. Rillo, Karen Robinson, Takuya Sagawa, Francisco Sierro, Kunio T. Takahashi, Adi Torfstein, Igor Venancio, Makoto Yamasaki, Patrizia Ziveri

**Affiliations:** 1 Aix-Marseille Université, CNRS, IRD, INRAE, CEREGE, Aix-en-Provence, France; 2 Department of Climate Geochemistry, Max Planck Institute for Chemistry, Mainz, Germany; 3 Fondation pour la recherche sur la biodiversité (FRB-CESAB), Montpellier, France; 4 Université Littoral Côte d’Opale, Univ. Lille, CNRS, UMR 8187, LOG, Laboratoire d’Océanologie et de Géosciences, Wimereux, France; 5 NIOZ, Royal Netherlands Institute for Sea Research, Department of Ocean Systems, Texel, The Netherlands; 6 Institute of Oceanology, Polish Academy of Sciences, Sopot, Poland; 7 Mercator Ocean International, Toulouse, France; 8 LPG-BIAF, UMR-CNRS 6112, University of Angers, Angers, France; 9 MARUM, Center for Marine Environmental Sciences, University of Bremen, Bremen, Germany; 10 Tohoku University Museum, Tohoku University, Miyagi, Japan; 11 BRIDGE, School of Geographical Sciences, University of Bristol, Bristol, UK; 12 Universitat Autonoma de Barcelona, ICTA and Dept. of Geography, Barcelona, Spain; 13 Geological Survey of Israel, Jerusalem, 9692100 Israel; 14 Fukui Prefectural Satoyama-Satoumi Research Institute, 22-12-1, Torihama, Wakasa, Mikatakaminaka, Fukui, 919-1331 Japan; 15 Israel Oceanographic & Limnological Research, Haifa, 31080 Israel; 16 Laboratoire des Sciences Du Climat et de L’Environnement, Domaine Du CNRS, Gif-sur-Yvette, 91198 France; 17 Department of Marine, Earth, and Atmospheric Sciences, North Carolina State University, Raleigh, NC USA; 18 Department of Natural and Computational Sciences, Hawaii Pacific University, Kaneohe, HI 96744 USA; 19 Department of Earth Science, Geological Institute, ETH Zürich, Zürich, Switzerland; 20 SCAR life Sciences. Formerly of the Australian Antarctic Division, Department of the Environment, 203 Channel Highwa, Kingston, Tasmania 7050 Australia; 21 Climate Change Institute, The Australian National University, Canberra, Australian Capital Territory Australia; 22 GEOMAR Helmholtz Centre for Ocean Research Kiel, 24148 Kiel, Germany; 23 The Marine Biological Association,The Laboratory, Citadel Hill Plymouth, Devon, PL1 2PB UK; 24 Woods Hole Oceanographic Institution, Woods Hole, MA 02543 USA; 25 Australian Antarctic Division, Department of Climate Change, Energy, Environment and Water, Kingston, 7050 Tasmania Australia; 26 School of Resource and Environmental Management, Simon Fraser University, Burnaby, Canada; 27 School of Environmental Science, Simon Fraser University, Vancouver, Canada; 28 Programa de Pós-Graduação em Geoquímica Ambiental, Universidade Federal Fluminense, Niterói, 24.020-141, Rio de Janiero, Brazil; 29 British Antarctic Survey, High Cross, Madingley Road, Cambridge, CB30ET UK; 30 Departamento de Zoología, Universidad de Concepción, Concepción, Chile; 31 Centre for Arctic Gas Hydrate, Environment and Climate, Department of Geosciences, UiT, The Arctic University of Norway, Tromsø, Norway; 32 College of Oceanic and Atmospheric Sciences, Oregon State University, Corvallis, USA; 33 Geoscience Australia, GPO Box 378, Canberra, ACT 2601 Australia; 34 Universidad de Salamanca, Geology Department (Paleontology), Salamanca, Spain; 35 ICBM, Institute for Chemistry and Biology of the Marine Environment, University of Oldenburg, Wilhelmshaven, Germany; 36 NIWA, Riccarton, Christchurch, New Zealand; 37 Kanazawa University, Kakuma-machi, Kanazawa, Ishikawa, 9201192 Japan; 38 Departamento de Geología, Universidad de Salamanca, 37008 Salamanca, Spain; 39 National Institute of Polar Research, Tachikawa, Japan; 40 The Fredy & Nadine Herrmann Institute of Earth Sciences, The Hebrew University of Jerusalem, Jerusalem, 91904 Israel; 41 Interuniversity Institute for Marine Sciences, Eilat, 88103 Israel; 42 Programa de Geociências (Geoquímica), Universidade Federal Fluminense, Niterói, Brazil; 43 Department of Earth Resource Science, Graduate school of International Resource Sciences, Akita University, 1-1 Tegata-Gakuencho, Akita, 010-8502 Japan; 44 Catalan Institution for Research and Advanced Studies (ICREA), Barcelona, Spain

**Keywords:** Biodiversity, Climate change

## Abstract

Planktonic Foraminifera are unique paleo-environmental indicators through their excellent fossil record in ocean sediments. Their distribution and diversity are affected by different environmental factors including anthropogenically forced ocean and climate change. Until now, historical changes in their distribution have not been fully assessed at the global scale. Here we present the FORCIS (Foraminifera Response to Climatic Stress) database on foraminiferal species diversity and distribution in the global ocean from 1910 until 2018 including published and unpublished data. The FORCIS database includes data collected using plankton tows, continuous plankton recorder, sediment traps and plankton pump, and contains ~22,000, ~157,000, ~9,000, ~400 subsamples, respectively (one single plankton aliquot collected within a depth range, time interval, size fraction range, at a single location) from each category. Our database provides a perspective of the distribution patterns of planktonic Foraminifera in the global ocean on large spatial (regional to basin scale, and at the vertical scale), and temporal (seasonal to interdecadal) scales over the past century.

## Background & Summary

Planktonic Foraminifera are marine unicellular eukaryotes with calcareous shells and chambered tests. Building on the classical pioneering works of Bradshaw^1^, Bé and Tolderlund^2^, and Bé^3^, planktonic Foraminifera (phylum of the Rhizaria supergroup) contain about 50 extant morphospecies in the global ocean^4–7^. Planktonic Foraminifera are sensitive to environmental conditions, many of which are registered by the chemical composition of their calcareous shells. As a result, their fossil record is widely used to reconstruct paleo-environments^8–10^.

Understanding the impacts of climate change on planet Earth and its ecosystems, especially the vast expanses of the surface ocean, is a global challenge and thus central to many ecological and biogeochemical studies^11–14^. Until now, the impacts of anthropogenic stressors on the distribution and biodiversity of planktonic organisms are poorly understood at the global scale^14^. Hence, better knowledge of the role of multiple stressors on the dynamics of modern planktonic communities and observational data on the distribution and biodiversity in the global ocean are required to assess past, present, and future developments of the marine ecosystem in response to expected changes of the global marine environment^15–17^. Most of the planktonic Foraminifera species live between the surface and the seasonal thermocline of the open ocean^5,18,19^, exposing them to a multitude of stressors including anthropogenic effects such as ocean acidification. Ongoing global warming combined with chemical changes in ambient seawater is affecting their calcification, biodiversity, and distribution at the community levels^20–24^.

As a ubiquitous but minor part of the total marine biomass^25^, planktonic Foraminifera serve as a model for pelagic biodiversity studies^26,27^, though their potential has been mostly explored in paleoenvironmental studies^28^. The FORCIS project evaluates changes in the diversity, distribution and abundance of planktonic Foraminifera (vertical and horizontal) in response to multiple climatic stressors by compiling data on samples from water column at the global scale^29^.

**Fig. 1:**
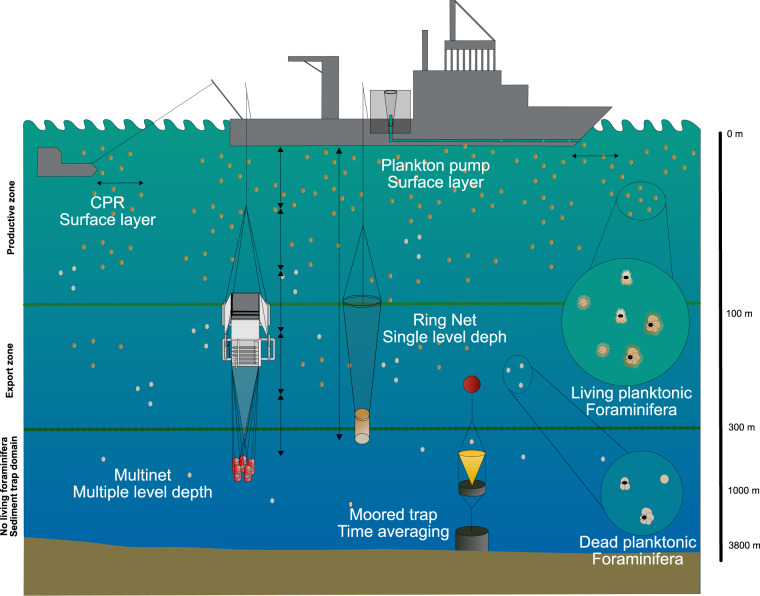
Schematic representation of the sampling devices deployed to collect modern planktonic Foraminifera from the global ocean at different depth levels, from a “snapshot” to an averaged time record, and integrated into the FORCIS database. CPR and plankton pump are sampling mainly the living planktonic Foraminifera living (yellow dots) in the upper ocean. The sediment trap is collecting mainly dead Foraminifera fluxes (white dots). The plankton net and multinet are sampling larger depth ranges. Arrows indicate resolution of the depth level(s).

**Fig. 2:**
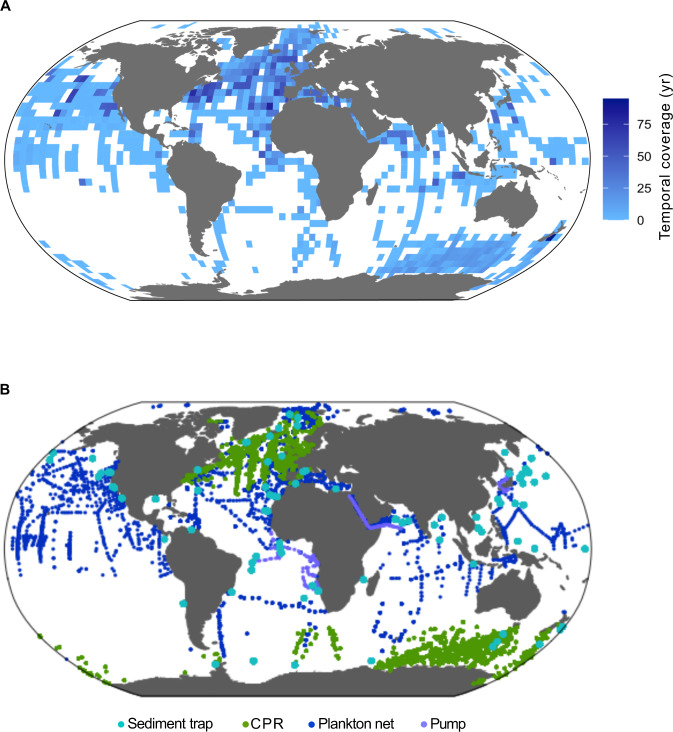
(**A**) Temporal and spatial coverage of the FORCIS data at 4 × 4 degree (latitude and longitude) grid resolution colored for the time series range (years) of each cell. (**B**) Geographical locations of all records included in the FORCIS database.

The FORCIS database contains data on planktonic Foraminifera abundance in the global ocean from plankton tow, Continuous Plankton Recorder (CPR), plankton pump, and sediment trap samples, and is meant to provide a synoptic view from the earliest observations in 1910 until 2018 (Figs. 1, 2). These data are based on physically extracted organisms rather than *in situ* imaging techniques. Data obtained from plankton nets, plankton pumps and CPRs take “snapshots” of the distributions of both living and dead Foraminifera species in the water column, while data obtained from sediment traps are “time integrators” of mostly fluxes of dead individuals (tests) settling from the surface ocean. The CPR and plankton pump collecting techniques mainly sample surface waters to about 10 m depth^30,31^, while oblique or vertical towed plankton nets, such as the commonly used Multinet (e.g., Schiebel^12^), sample the productive water column to the export zone (hundreds of meters) over single or multiple depth levels (Fig. 1).

The data presented in the FORCIS database aim to improve our understanding about (1) potential spatial and vertical migrations, (2) phenology, and (3) the various of effects global climate change on the planktonic Foraminifera biogeography, as well as their vertical and seasonal distribution over the past decades. Because of the temporal range of the observations, the database can also be used to investigate the impact of anthropogenic ocean change on planktonic Foraminifera distribution and ecology.

## Methods

### Data collection

The database currently includes 188,000 planktonic Foraminifera subsamples. Each subsample represents one aliquot of planktonic foraminifera specimens collected within a specific depth range, time interval, size fraction range or identified as living or dead, at a single location sampled via plankton pumps and nets, CPR and sediment traps (more details in the Data Records section). The compiled data were gathered from published scientific literature, PhD or master’s theses, books, databases, unpublished datasets, and reports. Some data were directly provided by contributors from the FORCIS working group and their personal networks. The dataset includes contributions from around 140 published and unpublished references^1,23,32–168^ reporting on the diversity and distribution of planktonic Foraminifera, spanning a time interval of more than a century (1910–2018). Most of the datasets published before 1960 were digitized from tables or plots from dissertations (some available as a hardcopy only) and scientific papers, or sourced from the contributor’s digital data files. Data on planktonic Foraminifera were extracted manually or automatically using WebPlotDigitizer-4.3 software^169^, including number concentrations of tests (ind/m^3^), relative numbers (%), and fluxes (ind/m^2^/day) collected from the global ocean using plankton nets, CPR, plankton pumps, and sediment traps (Table 1 and Fig. 2A,B). Moreover, data indicating only the presence or the absence of species were also retrieved. Binned data of species (i.e., estimated number concentrations or percentage concentrations reported for a minimum and maximum depth range like for the CPR data in the North Atlantic) were also collected and included in the FORCIS database. Foraminifera abundance data are divided into four categories, i.e., raw values (numbers of individuals), number concentrations (ind/m^3^), percentage concentrations (%), or fluxes (ind/m^2^/day).

**Table 1:** Temporal and spatial coverages of the planktonic Foraminifera samples in the FORCIS database and number of primary keys in the tables sites, profiles, samples, and subsamples.

Sampling device type	Spatial coverage		Temporal coverage	Number sites (site_id)	Number profiles (profile_id)	Number samples (sample_id)	Number subsamples (subsample_id)
	Latitude (°)	Longitude (°)					
Plankton tows	64° S/86° N	180° W/180° E	1910–2017	2176	2635	6216	21898
Sediment traps	65° S/77° N	177° W/179° E	1978–2018	101	129	4838	8908
CPR	77° S/79° N	180° W/180° E	1991–2018	6972	6972	157166	157166
Pump	22° S/53° N	39° W/143° E	1985–2008	282	291	291	384

### Database design and architecture

**Fig. 3:**
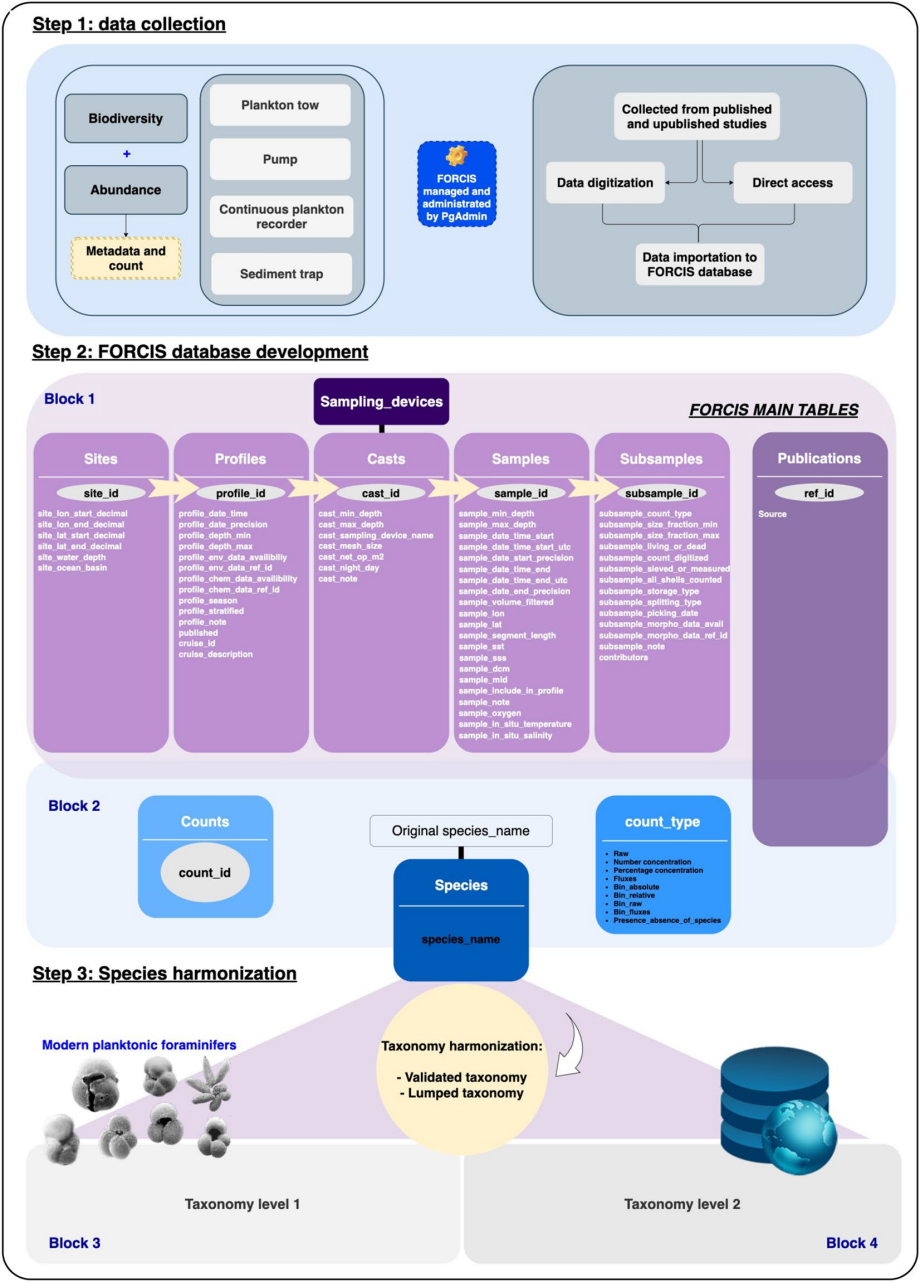
Methodology and structuring FORCIS database compilation: from data collection and different access levels to the final published database.

The FORCIS database is composed of ten tables with the counts of modern planktonic Foraminifera and metadata (Table 2), built and designed using PostgreSQL, which allows filtering and quality checking of the data during the importing and extraction steps. In the data tables, sites, profiles, casts, samples, subsamples, and counts are interconnected by five unique identifiers (primary keys) (Fig. 3) labeled as ‘site_id’, ‘profile_id’, ‘cast_id’, ‘sample_id’, and ‘subsample_id’. They present hierarchical levels and are automatically generated according to naming rules if not provided in the original dataset, during data import.

**Table 2:** FORCIS database main table and primary key description.

FORCIS table	Description
Sites Primary key: ‘site_id’ Number of variables: 7	Each site is characterized only by its location (longitude and latitude coordinates). Associated information are water depth and ocean basin. The unique identifier or primary key (‘site_id’) could be either sourced from the original publication/study (e.g. PECH_B), or generated by the database managers (e.g. MedSeaCruise_St1).
Profiles Primary key: ‘profile_id’ Foreign key (s): ‘site_id’ Number of variables: 15	For the net data, profiles are distinguished by their time of collection and location as well. Overall, profile_id’s have the same coordinates, and different times of sampling were incremented. For the sediment trap data, the profile_id is incremented when the deployment has changed. The date, depth range of each profile, availability of the environmental data including ambient seawater chemistry, and profile season are also included in the table profiles.
Casts Primary key: ‘cast_id’ Foreign key (s): ‘site_id’ and ‘profile_id’ Number of variables: 8	Provides information regarding the casts, the sampling device name, depth range of a cast, mesh size, and net opening of the plankton tow.
Samples Primary key: ‘sample_id’ Foreign key (s): ‘site_id’, ‘profile_id’ and ‘cast_id’ Number of variables: 22	Each sample in this table is characterized by its depth range, volume of water filtered (for net data), coordinates, segment length (for CPR data), date of sampling, and *in situ* temperature and salinity.
Subsamples Primary key: ‘subsample_id’ Foreign key (s): ‘site_id’, ‘profile_id’, ‘cast_id’ and ‘sample_id’ Number of variables: 15	One sample could be divided into different subsamples (‘subsample_id’) based on their subsample_size_fraction_min, subsample_size_fraction_max or/and subsample_living_or_dead. Other information is also reported in this table such as: subsample_count_type, subsample_sieved_or_measured, subsample_storage_type, and subsample_splitting_type.
Counts Identifier: species_name Foreign key (s): ‘site_id’, ‘profile_id’, ‘cast_id’, ‘sample_id’ and ‘subsample_id’	The table contains counts per species or total number of species for each subsample. A count value is made unique by its subsample_id and the species name.
Publications Primary key: ‘ref_id’ Number of variables: 2	The publications table covers all references of publications and studies published or unpublished from where the data were sourced. A ref_id was assigned to each reference and listed in this table.
Species Number of variables: 3	The different species’ original names were kept in the FORCIS database as they were given by the data contributor or in publication/manuscript and listed in the table species. This species names list was formatted according to 2 levels of taxonomy: level 1 “validated taxonomy” and level 2 “lumped taxonomy”.
Sampling_devices Number of variables: 3	There are only four main sampling device types comprising this table, which are: net (plankton tow), CPR, sediment traps, and pump.
Count_type Number of variables: 2	Count data in FORCIS are reported in different units: raw as number of individuals, absolute (ind/m^3^), relative (%) and fluxes (ind/m^2^/day). The binned data were also included in the FORCIS database as a range of values (raw as number of individuals, absolute (ind/m^3^), relative (%) or fluxes (ind/m^2^/day)).

Each site (‘site_id’) is characterized by its geographic information (coordinates in longitude and latitude form). Associated information includes water depth and the name of the ocean basin. Data from the same site are separated into different profiles (‘profile_id’) according to their collection time (“profile_date_time”). Profiles are made of different casts, which are defined by their sampling device and depth. Each cast (having a unique ‘cast_id’) contains at least one sample, which is given a unique sample ID (‘sample_id’) based on their sampling time and depth. Subsamples with unique IDs are used to distinguish samples from different size fractions (size_fraction, min and max), and living or dead specimens when available. Finally, each individual count value is identified by its species name, and belongs to a unique set of ‘subsample_id’, ‘sample_id’, ‘cast_id’, ‘profile_id’ and ‘site_id’. Each line in the database is assigned to a reference (‘ref_id’), for example, a published paper, manuscript, book, or unpublished study with information on the data contributor. Moreover, the FORCIS database comprises information regarding the sampling methodology (i.e., sampling device, mesh size), and nature of the subsamples where applicable, for example, distinction between individuals with filled or empty tests (often referred to as “dead specimens”).

**Fig. 4:**
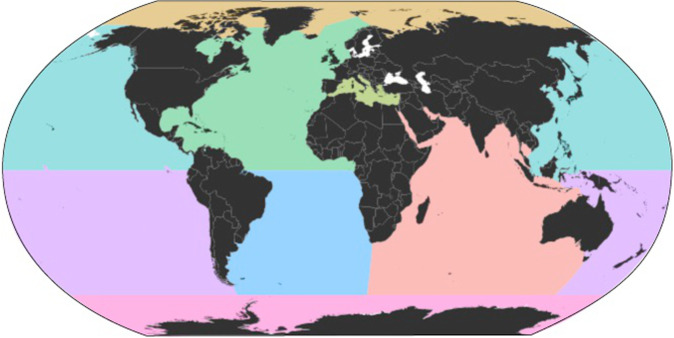
Oceanic basin boundaries defined by the International Hydrographic Organization (IHO) database map. Note that white areas were excluded.

Eight major ocean basins are distinguished, the Arctic, Antarctic, South Pacific, North Pacific, South Atlantic, North Atlantic, Indian Ocean, and the Mediterranean Sea using QGIS software (3.16 Hannover; available online https://www.qgis.org/es/site/), whose boundaries were defined by adapting the shapefile published by the International Hydrographic Organization (IHO) database map^170^ (Fig. 4). Each sampling site (‘site_id’) in the FORCIS database is associated with its respective oceanic basin.

### Importing the datasets

The metadata and count data were collected using a spreadsheet template (available at Zenodo^171^). Several variables in this template are mandatory fields to make sure that related tables of the database can be filled and linked together, i.e., for each hierarchical level: site coordinates (‘site_lon_start_decimal’, ‘site_lat_start_decimal’); profile date (‘profile_date_time’); cast information (‘cast_min_depth’, ‘cast_max_depth’, ‘cast_sampling_device_name’); sample information (‘sampling_device_type’, ‘sample_min_depth’, ‘sample_max_depth’, ‘sample_date_time_start’); subsample information (‘subsample_count_type’, ‘subsample_size_fraction_min’), and bibliographic information (either full reference or ‘doi’ for recent datasets).

For data safety, updates of the FORCIS database were routinely saved under different versions and stored in a SQL server. In parallel, data quality control and curation were done during the database development to ensure maximum quality consistency.

### Database harmonization, curation, and quality control

All dataset entries underwent a series of quality control, curation, harmonization, and standardization steps, during and after inclusion in the database, in close collaboration between database managers and data contributors. For example, ‘site_lon_start_decimal’ and ‘site_lat_start_decimal’ were quality controlled and checked, for example, for redundancies and inconsistencies to avoid replicating datasets. Imported data were first screened by the database managers to check for inconsistencies such as negative depth range (i.e., minimum depth larger than maximum depth), and second by the members of FORCIS working group, to apply quality control and minimize the errors. Different maps were generated to validate the geographical data distribution, check and correct our entries for outliers. For example, maps of species distributions were produced to check for regional and ecological plausibility that helped quality check the dataset for mistyping while assembling the data and for the taxonomy harmonization. However, none of the data retrieved from the original publication was corrected or excluded. Species counting information distinguishes the absence of information (NA, Not Available) from the absence of the specimens (value of zero).

### Harmonization of the taxonomy

Species names were initially kept in the database as given by the data contributor or by the original publication, with minor corrections being made for spelling errors. Genus attributions had to be harmonized to a common standard^5,7,172^, to ensure that each taxon is labeled in the database with a unique binomen or trinomen. Finally, abbreviations and names referring to further attributes that can be taxonomically significant (shell pigmentation, coiling direction) were also harmonized to a common standard, to facilitate automated analysis. The resulting list of harmonized binomina or trinomina with harmonized names of additional attributes (original taxonomy) was used to resolve two further taxonomic issues: synonymy (different names given to the same underlying taxon) and shifting taxonomic concepts (splitting or lumping, including new taxa). Both issues result from the fact that formal taxonomy always reflects the opinion of the author and is subject to change as new knowledge emerges. The resulting “validated taxonomy” contains 55 species and categories, preserving in a consistent manner information that may be taxonomically relevant, but is presently not reflected in formal nomenclature (coiling direction, presence of specifically shaped terminal chambers).

Since 1960, new species have been described that were not recognised before or lumped with others (e.g., *Neogloboquadrina incompta*^173^*, Globorotalia eastropacia*^174^*, Berggrenia pumilio*^175^*, Globigerinella calida*^176^*, Globorotalia cavernula*^176^*, Globorotalia ungulata*^177^*, Orcadia riedeli*^178^*, Tenuitellita fleisheri*^179^). As such additional information is not always provided, the validated taxonomy has been subsequently mapped onto a “lumped taxonomy” comprising 46 species and categories that could be recognized in all datasets. The names used for all formally described taxa follow Schiebel and Hemleben^5^ and references therein, as expanded by Morard *et al*.^172^, and revised by Brummer and Kucera^7^ and references therein.

In most cases, the mapping of synonyms onto the validated taxonomy and the contraction of the validated taxonomy onto the lumped taxonomy was straightforward and the procedure can be understood directly from the synonym lists provided (available at Zenodo^171^). There are two notable exceptions, which require explanations. The first concerns the treatment of coiling variants in the abundant and variable genus *Neogloboquadrina*. In the high latitudes, oppositely coiled *N. pachyderma* have been often, but not always, recognized and counted separately. Darling *et al*.^180^ confirmed that the coiling variants represent different genetically distinct lineages, so that sinistral specimens are assigned to *N. pachyderma* and dextral specimens to *N. incompta*. Where coiling direction was not recorded, the counts are reported as the sum of both species (n_pachyderma_any). The second exception concerns the species *Globigerinoides ruber*, where the presence of pink- and white-pigmented specimens and the erroneous synonymization of *G. elongatus* with *G. ruber* resulted in complex and often ambiguous taxonomic attributions. The nomenclature of *G. ruber* in the FORCIS database follows the concept of Morard *et al*.^172^, with pink-pigmented specimens, when counted separately, being named *G. ruber ruber*, and non-pigmented specimens being attributed either to *G. ruber albus* with inflated chambers, or *G. elongatus* with compressed chambers. Where the distinction between *G. ruber albus* and *G. elongatus* has not been made, the counts are reported as the sum of both species (g_ruber_albus_or_elongatus). In cases where not even the shell pigmentation has been considered, we only report the count for all three categories together (g_ruber_any).

### Extracting data

The hierarchical structure of the database, split into different related tables, facilitates swift extraction of large merged data volumes. It is possible to retrieve count data and/or metadata separately and to apply filters to extract specific sub-datasets.

As the SQL was only used to develop and quality check the FORCIS database, the finalized version of the database was extracted from the SQL and converted to “.csv” files and made available on Zenodo to facilitate the handling of the data for the users. To facilitate the handling of the database in the Zenodo “.csv” files, an R-package was compiled (https://frbcesab.github.io/forcis/), providing basic functions to extract the data from the different files based on different taxonomy levels and harmonize the species counts into a unique count type.

In the final published database, all data coming from different sampling devices were put into separate “.csv” files. Only the data of the CPR from the Southern Hemisphere have been separated from those CPR data collected from the Northern Hemisphere as the data structure is different (species-level resolved counts vs. binned total counts, respectively). Each of the 5 “.csv” files contain metadata and original species counts.

Updates on the last database versions will be released in csv format. We foresee a continuous update of the database depending on the number of new datasets published. The labels of updated versions of the released “.csv” files will contain the date of their publication and versioning number.

## Data Records

The FORCIS database is published as five “.csv” files composed of data from four types of sampling devices, i.e., plankton tows, plankton pump, CPR (“.csv” file for each data from the Southern and from the Northern Hemispheres), and moored sediment traps, and the associated dataset is uploaded on the Zenodo repository^171^ (Fig. 2). These files encompass more than 188,000 subsamples including ~157,000 CPR (since 1991), ~22,000 net (since 1910), ~9,000 sediment trap (since 1978), and 400 pump (since 1985) subsamples (Table 1).

The data in FORCIS are presented as follows: each row in the database is a subsample *(i.e*., one single plankton aliquot collected within a water depth range, time interval, size fraction, at a single location) associated to 1) “**block 1**”: the metadata (*i.e*., location, date, depth, cast, environmental data of this record), and 2) “**block 2**”: the original data as reported in the data sources (abundance and/or diversity). The FORCIS database metadata has a hierarchical structure (Fig. 3): first, all sites are assigned to a **site_id** associated with the coordinates (**site_lon_start_decimal** and **site_lon_end_decimal**) and **site_ocean_basin**. Then, for each profile collected at the different site, a **profile_id** is attributed, based on the **profile_date_time** (time of the collection) and coordinates (Table 2). The depth range (**profile_depth_min** and **profile_depth_max**) of each profile, and environmental data including ambient seawater chemistry (**profile_env_data_availability** and **profile_chemical_data_availability)**, and **profile_season** are given. Information regarding the different **cast_id** used for each profile_id is provided in the metadata block, such as: **cast_sampling_device_name,** **cast_min_depth,** **cast_max_depth,** **cast_mesh_size** of the plankton tow. For each individual sample, a **sample_id** is assigned, including depth range (**sample_min_depth** and **sample_max_depth**), **sample_volume_filtered** (for net data), coordinates (**sample_lon** and **sample_lat**), **sample_segment_length** (for CPR data), date of sampling (**sample_date_time_start** and **sample_date_time_end**), and *in situ* temperature and salinity data (**sample_in_situ_temperature** and **sample_in_situ_salinity**). Each sample can be divided into different **subsample_id** based on their size **(subsample_size_fraction_min,** **subsample_size_fraction_max)** and/or filled or not tests (**subsample_living_or_dead)**. Other information is also reported in this table such as: **subsample_count_type,** **subsample_sieved_or_measured**, and **subsample_storage_type** and **subsample_splitting_type**. The contributors who provided the data are given in the column **contributors**, and the source of their data (**ref_id** and **source**) is reported for each subsample.

Each subsample is associated with its corresponding counts that could be either the abundance of a species or the total number of Foraminifera specimens (i.e., those not identified at the species level), and reported in the table **count**. The species names are kept as they were reported in the original data source and listed as species names in block 2.

Two taxonomic levels (level 1 “validated taxonomy” and level 2 “lumped taxonomy”) can be generated in two separated blocks (**block 3** for taxonomy level 1, and **block 4** for taxonomy level 2; Fig. 3).

## Technical Validation

The compilation of ~188,000 subsamples resulted in a high number of counts in the FORCIS database (more than 1,300,000 species counts and ~1,200,000 non-zero counts), compared to fossil planktonic Foraminifera databases such as ForCenS (~4,000 subsamples, and ~ 60,000 counts) that reports data of the planktonic Foraminifera found in the surface sediment samples^181^. The *Triton* database^182^ holds ~500,000 non-zero counts of planktonic Foraminifera occurrences during the Cenozoic. However, the FORCIS database holds a lower number of samples compared to the COPEPOD database (~400,000) which is a global-coverage database of zooplankton abundance, phytoplankton abundance, and zooplankton biomass data^183^.

**Fig. 5:**
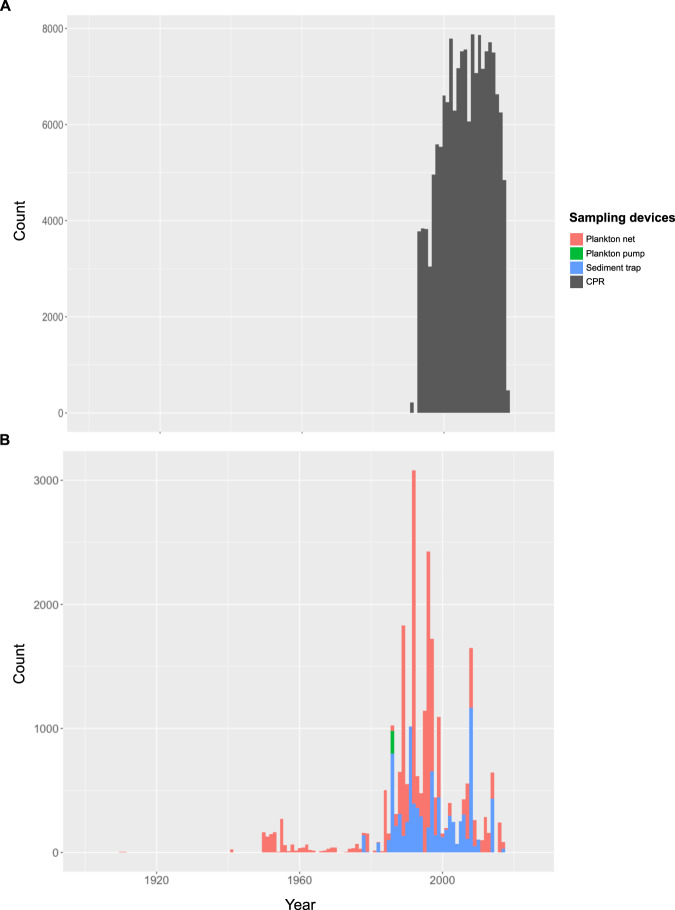
Number of subsamples collected by CPR (**A**), plankton net, plankton pump and sediment traps (**B**) per year in the FORCIS database.

Temporal data coverage varies temporally and spatially, but is highest after 1990 and in the Northern Hemisphere (Fig. 5). The plankton net dataset presents the widest temporal (from 1910 until 2017), and spatial ranges (from 61° S to 86° N, and 180° W to 180° E, Table 1). The sediment trap dataset includes data from 1978 to 2018, from 65° S to 77° N, and 177° W to 179° E. The CPR dataset covers the subtropical to polar oceans, from 30° N to 79° N, and 79° W to 20° E in the Northern Hemisphere, and from 77° S to 40° S, and 180° W to 180° E in the Southern Hemisphere. All CPR samples included here^30,31^ were collected during a time period from 1991 to 2018 (Fig. 5A). The pump dataset has the smallest regional coverage ranging from 22° S to 53° N, and 39° W to 143° E.

Despite more than a century of work, large parts of the ocean have remained unsampled for planktonic Foraminifera, *e.g*., the Southern Pacific Ocean (Fig. 2). The temporal coverage of the FORCIS database exposes a low sampling effort especially during the time period before 1960, with only ~1,000 subsamples collected between 1910 and 1960 (Fig. 5B). In addition, few datasets are available from certain seasons, such as winter data from high latitudes due to the lack of sampling campaigns.

The FORCIS database comprises an extensive coverage of the Northern Hemisphere (Fig. 2A), especially of the North Atlantic Ocean. In contrast, plankton tows and sediment traps from the Southern Ocean are sparse due to difficulties associated with sampling in remote and stormy regions. However, despite these temporal and spatial gaps, the amount of data in FORCIS covers broad swaths of the global ocean and facilitates comparison of changes in distribution and diversity within and between different provinces over time (Fig. 2B).

Although FORCIS contains fewer species per sample than the coretop synthesis ForCenS (6 vs. 15), it contains more species than ForCenS when using the same taxonomic level in both databases, i.e., 46 vs. 40 species, respectively. The main reason for this difference is the coarser size fraction in ForCenS, which is limited to ≥150 μm^181^ vs. the finer size fractions in FORCIS that extend down to 30 μm; only these latter finer size fractions include small-sized species such as *S. globigerus, N. vivans, O. riedeli, T. clarkei, T. fleisheri* and *T. parkerae*, which are not included in ForCenS^181,184^.

Moreover, more species are documented in FORCIS compared to core-top sediment databases (e.g., CLIMAP^185^, Brown Foraminiferal Database^186^, ForCens), and the use of species names is not fully complementary between this study and the earlier databases. In addition, thin shells of small-sized species such as *O. riedeli* and *T. parkerae* may dissolve during settling in the water column before reaching the ocean floor and are therefore not present in ForCenS^187,188^.

## Usage Notes

Filtering of data in the FORCIS database allows the user to select particular datasets (*e.g*., by latitude, longitude, season, ocean basin, year). Seasons were distinguished between the Northern Hemisphere (defining Autumn by September, October, November; Winter by December, January, February; Spring by March, April, May and Summer by June, July, August) and Southern Hemisphere (defining Spring by September, October, November; Summer by December, January, February; Autumn by March, April, and Winter by June, July, August). The type of original count data was kept in FORCIS as reported in the original study (raw, number concentration (ind/m^−3^), percentage concentration (%), bin or fluxes). Data are presented as counts of the identified specimens or total abundance of all the species found in the sample including unidentified specimens. In the latter case, the count is reported in the column **unidentified_specimens**.

In most cases, the number concentration is given by the data contributors, in others, the sampled volume of seawater could be calculated for vertical tows using the surface area of the net times the depth interval. When the total number of Foraminifera or volume of sampled seawater are not provided, the number concentration cannot be calculated (see column on **subsample_absolute_abundance_available**). The number concentration reported in FORCIS are raw numbers corrected for split and the filtered volume when available, but are not standardized for either the mesh size or sieve size fraction. This is important since different sizes will significantly affect number and percentage concentrations (e.g., Berger, 1969).

The column **subsample_count_type** gives the type of count reported in the database. All the counts reported as 0 (zero) in the original study were kept in the FORCIS database, which means that the respective species was not found in the sample. However, the absence of species has not always been consistently recorded because of different counting procedures (e.g., researchers working in the polar areas have not consistently reported the absence of tropical species). To express this, the column **subsample_all_shells_present_were_counted** helps the user to identify in which datasets a species may have been present but was not counted. For subsamples with “complete” taxonomic coverage, the entry in this column is “true”.

All counts without clear location (nine subsamples) and/or date of sampling (274 subsamples) were kept in the database even though they cannot be used directly for spatial and time-series analyses. A note has been associated with the corresponding subsamples.

The **number_of_species_counted** was calculated when all the species were counted in the subsample and provided for both levels of taxonomy (in block 3 and block 4) based on the number of planktonic Foraminifera species observed in each subsample. The number of **benthic** species was included in FORCIS when given in the original data source but is not included in the calculation of Foraminifera diversity.

Finally, the FORCIS database will be open for any new data entry, and the FORCIS project warmly welcomes any new data published or provided by any contributor by submitting the data through our website (https://forcis.cerege.fr/).

## Data Availability

No custom code was used.
